# Memory complaints, clinical aspects, and self-esteem in adult people with epilepsy

**DOI:** 10.1590/1980-57642021dn15-030007

**Published:** 2021

**Authors:** Glória Maria de Almeida Souza Tedrus, Laura Annoni Lange

**Affiliations:** 1 Postgraduate Program in Health Sciences, Pontifícia Universidade Católica de Campinas – Campinas, SP, Brazil.; 2 School of Medicine, Pontificia Universidade Católica de Campinas – Campinas, SP, Brazil.

**Keywords:** epilepsy, memory, memory complaint questionnaire, self-esteem, epilepsia, memória, questionário de queixa de memória, autoestima

## Abstract

Memory complaints are common in adult people with epilepsy (PWEs). However, the associated clinical aspects are not yet fully understood.

**Objective:**

This study aims to relate the occurrence of memory complaints in PWEs with clinical aspects and self-esteem.

**Methods:**

To relate the data obtained from the Memory Complaint Questionnaire (MAC-Q) with clinical aspects, 71 PWEs were assessed using the Rosenberg Self-Esteem Scale (SES), the Mini Mental State Examination (MMSE), and the Brief Cognitive Battery-Edu. These data were compared with 55 individuals in a control group (CG).

**Results:**

Memory complaints (MAC-Q≥25) were significantly higher in PWEs, when compared with individuals in the CG [35 (49.3%) vs. 15 (27.2%); Student’s *t*-test; p=0.012]. Objective cognitive performance was lower in PWEs. Memory complaints were associated with a lower educational level, the presence of depression, SES, MMSE, incidental memory, and the clock-drawing test scores in PWEs.

**Conclusions:**

Memory complaints were more frequent in PWEs than in individuals in the CG, and there was a relationship with cognitive deficit, educational level, depression, and low self-esteem.

## RESUMO

Queixas de memória são frequentes em pessoas adultas com epilepsia (PCEs). Entretanto, os aspectos clínicos associados ainda não são totalmente compreendidos.

**Objetivo:**

Relacionar a ocorrência de queixas de memória em PCEs com aspectos clínicos e a autoestima.

**Métodos:**

Relacionar os dados do Memory Complaint Questionnaire (MAC-Q) com aspectos clínicos, a Roserberg Self-esteem Scale (SES), o MEEM e a BCB-Edu de 71 PCEs. Os dados foram comparados aos de 55 indivíduos normais (GC).

**Resultados:**

Queixa de memória (MAC-Q≥25) foi significativamente maior nos PCEs quando comparada a do GC [35 (49,3%) vs. 15 (27,2%); teste t de Student; p=0,012]. O desempenho cognitivo objetivo foi inferior nas PWEs. As queixas de memória foram associadas à menor escolaridade, presença de depressão e à SES, o MEEM, à memória incidental e o clock-drawing test nas PCEs.

**Conclusões:**

As queixas de memória foi mais frequente nas PCEs do que no GC, e houve relação com o déficit cognitivo, a escolaridade, a depressão e a baixa autoestima.

## INTRODUCTION

The presence of self-perceived memory problems is quite common in the general population, increasing with aging,^1^ ^,^ ^2^ with some studies describing a relationship between memory complaints and a higher risk of progressing to dementia.^3^ ^,^ ^4^ ^,^ ^5^ ^,^ ^6^ However, it remains controversial whether memory complaints accurately are actually related to objective performance in cognitive tests.^7^ ^,^ ^8^ ^,^ ^9^

In epilepsy, objective cognitive deficits and memory complaints are frequent.^10^ ^,^ ^11^ ^,^ ^12^ Between 20 and 50% of adult people with epilepsy (PWEs) complain of poor memory.^13^ Memory difficulties is one of the aspects of epilepsy that mostly compromises social functioning, self-esteem, and quality of life.^14^ ^,^ ^15^ In contrast, some studies suggest that PWEs overreported memory difficulties.^16^

Studies on cognitive assessments are frequent in the elderly people; however, the neurophysiological and clinical aspects associated with memory complaints in adult PWEs are not yet fully understood.

Therefore, assessing the occurrence of memory complaints in PWEs and investigating the relationship with cognitive performance, clinical aspects, and self-esteem are fundamental for advancing treatment options and solutions.

## METHODS

This is a cross-sectional study in which 71 individuals diagnosed with epilepsy were assessed according to the criteria of the International Classification of Epilepsies and Epileptic Syndromes (ILAE).^17^ The inclusion criteria were individuals (of both sexes) diagnosed with epilepsy, aged between 18 and 60 years, and attended at the neurology outpatient clinic of the PUC-Campinas University Hospital. Individuals with difficulties in understanding the instruments and those with progressive neurological diseases and sequelae of stroke and head trauma were excluded.

A control group (CG) was formed, consisting of 55 adults with no history of neurological and psychiatric diseases, or any other chronic disabling diseases, and similar in socioeconomic conditions, age, and educational level.

The Human Research Ethics Committee of PUC-Campinas approved the study. Clinical and demographic data were collected. The PWEs underwent neurological investigation with a detailed medical history and the collection of clinical data such as age of onset, type, and frequency of seizures and antiepileptic drugs (AEDs) in use. Complementary exam data were collected from the hospital’s medical records. The assessment of the presence of depression was carried out in the psychiatric service according to the criteria of the Diagnostic and Statistical Manual of Mental Disorders (DSM-IV) and using the International Statistical Classification of Diseases and Related Health Problems (ICD-10).

For the cognitive assessment, cognitive screening tests validated for the Brazilian population were applied, such as the Mini Mental State Examination (MMSE)^18^ ^,^ ^19^ and the Brief Cognitive Battery-Edu (BCB-Edu).^20^ For the assessment of self-esteem, the Rosenberg Self-Esteem Scale (SES)^21^ was used.

The Memory Complaint Questionnaire (MAC-Q)^22^ was used to assess the subjective complaints of memory impairment, which consists of six questions related to memory in the activities of daily living. The total score ranged from 7 to 35 points, and higher scores indicate a greater intensity of the complaint. The cutoff point established was ≥25.

### Data analysis

The Statistical Package for the Social Sciences software, version 22, was used for the statistical analysis of this study. The statistical significance was set at a p<0.05 in all tests.

The PWEs’ MAC-Q, MMSE, and BCB-Edu scores were compared with the CG’s scores. The PWEs’ MAC-Q scores were related to the clinical and cognitive aspects and to the SES scores.

Descriptive statistics were used to examine the sample characteristics and differences between groups. The Student’s *t*-test was used to compare the continuous variables and categorical variables.

## RESULTS

There was no significant difference in the sex, age, and educational level between PWEs and individuals in the CG (Table 1).

**Table 1.** Demographic, clinical, and cognitive data of people with epilepsy and individuals in the control group.

	PWE (n=71)	CG (n=55)	p-value
Sex: female	35 (49.2%)	33 (60%)	0.232^a^
Age (years)	52.4 (±6.1)	51.4 (±5.1)	0.318^b^
Educational level (years)	4.7 (±3.0)	5.5 (±2.0)	0.091^b^
MAC-Q (score)	25.2 (±5.4)	21.2 (±3.8)	0.001^b*^
MAC-Q>25	35 (49.3%)	15 (27.2%)	0.012^a*^
SES	22.4 (±6.0)	-	
MMSE (score total)	22.2 (±3.8)	25.3 (±2.4)	<0.001^b*^
BCB-Edu		
Identification	9.9 (±0.4)	9.9 (±0.1)	0.333^b^
Naming	9.8 (±0.6)	9.9 (±0.4)	0.331^b^
Incidental memory	5.6 (±1.4)	6.2 (±1.5)	0.019^b*^
Immediate memory	7.9 (±1.7)	8.9 (±1.0)	<0.001^b*^
Learning	6.9 (±1.7)	8.2 (±1.4)	<0.001^b*^
Recognition	8.9 (±1.0)	9.8 (±0.3)	<0.001^b*^
Clock-drawing test	10.7 (±4.1)	13.1 (±4.5)	<0.001^b*^
VF animals	5.2 (±2.7)	7.1 (±2.3)	0.008^b*^

PWEs: people with epilepsy; CG: control group; MAC-Q: Memory Complaint Questionnaire; SES: Rosenberg Self-Esteem Scale; MMSE: Mini Mental State Examination; BCB-Edu: Brief Cognitive Battery-Edu; VF animals: category fluency test. ^a^χ^2^ test; ^b^Student's *t*-test; *p<0.05.

The age of onset of seizures was 25.2 (±17.7) years. Seizures were exclusively focal in 35 (49.3%) patients, and they were focal to bilateral tonic-clonic or generalized onset in 36 (50.7%) patients. In the previous year, the frequency of seizures was ≥1 seizures/month in 25 (35.2%) cases and ≤11 seizures/year in 46 (64.7%) cases. Epileptic syndrome was classified as epilepsy of unknown etiology in 21 (29.6%) patients and as structural in 50 (70.4%) patients.

The MAC-Q scores were significantly higher in PWEs when compared with the CG. Objective cognitive performance was lower in PWEs when compared with the CG (Table 1).

In the CG individuals, a significant correlation was observed between BCB-Edu recognition and MAC-Q scores (Pearson’s correlation, r=0.377; p=0.005). No significant differences were found in the MAC-Q scores, according to the age and formal education, other cognitive aspects, and sex.

A greater memory complaint occurred in PWEs with a lower educational level (Table 2).

**Table 2.** People with epilepsy clinical and cognitive data and Rosenberg Self-Esteem Scale scores according to their Memory Complaint Questionnaire scores (<25 or ≥25)

	MAC-Q		p-value
	<25 (n=36)	≥25 (n=35)	
Sex: female/male	15/21	20/15	0.192^a^
Age (years)	53.1 (±4.8)	51.7 (±7.2)	0.354^b^
Educational level (years)	5.5 (±3.3)	3.9 (±2.5)	0.030^b*^
Epilepsy onset (age)	24.9 (±17.5)	25.4 (±18.1)	0.904^b^
Epilepsy duration (years)	28.1 (±16.4)	26.2 (±17.5)	0.641^b^
Seizure frequency: monthly/other	12/24	13/22	0.737
Depressive disorder: yes/no	10/26	20/15	0.012^b*^
SES	23.9 (±6.7)	20.9 (±4.8)	0.035^b*^
MMSE (score total)	23.3 (±3.3)	21.0 (±4.0)	0.010^b*^
Epileptic syndrome: unknown etiology/ structural	9/27	12/23	0.391
BCB-Edu		
Identification	10.0 (±0.0)	9.8 (±0.6)	0.169^b^
Naming	9.9 (±0.3)	9.7 (±0.7)	0.174^b^
Incidental memory	6.0 (±1.2)	5.3 (±1.5)	0.033^b*^
Immediate memory	7.6 (±1.9)	7.0 (±1.6)	0.208^b^
Learning	7.3 (±1.8)	6.8 (±1.7)	0.690^b^
Recognition	9.1 (±1.0)	9.0 (±1.0)	0.660^b^
Clock-drawing test	5.9 (±2.7)	4.6 (±2.6)	0.046^b*^
VF animals	11.1 (±3.9)	10.7 (±4.4)	0.693^b^

PWE: people with epilepsy; MAC-Q: Memory Complaint Questionnaire; SES: Rosenberg Self-Esteem Scale; MMSE: Mini Mental State Examination; BCB-Edu: Brief Cognitive Battery-Edu; VF animals: category fluency test. ^a^χ^2^ test; ^b^Student's *t*-test; *p<0.05.

The presence of subjective memory loss (MAC-Q≥25) was significantly associated with the presence of depression, with lower SES (low self-esteem), MMSE, incidental memory, and clock-drawing test scores. There was no significant difference in the MAC-Q according to the sex, type, and frequency of seizure; the number of antiepileptic drugs in use; and the epileptic syndrome (Table 2).

## DISCUSSION

This study observed a high occurrence of memory complaints in PWEs and had a significantly higher occurrence when compared with individuals in the CG. There was a significant relationship between the complaint and the presence of deficits in objective cognitive tests, with the presence of depression.

In objective cognitive tests, PWEs performed worse than individuals in the CG. The relationship between epilepsy and dysfunction in cognitive networks is well described in the literature,^10,12^ with several cognitive impairments, with the most common being memory.^23,24^

In the CG individuals, there was a correlation between the subjective and objective cognitive performances. In the literature, it was observed that memory complaints in adults are still poorly assessed. Studies in healthy elderly people show that memory complaint levels are high, more frequent in women, associated with the presence of depressive symptoms and with lower educational level, and highly sensitive for cognitive decline.^5,25,26^ However, other authors did not describe these associations.^1,27,28^

An association was observed between subjective memory complaints and deficits in objective tests in several areas, such as memory and visuospatial functions, as described in other studies in the literature.^16,29,30^ In a different way, some studies were unable to strongly relate subjective complaints with objective cognitive dysfunction and suggest the importance of investigating the neurophysiological process involved.^23,31^

Higher levels of memory complaints were associated with a lower educational level, which suggests the relationship between a lower initial cognitive status and greater cognitive vulnerability.^27^

Similar to other studies,^12,13,29,30,31,32^ a relationship was observed between poor subjective memory ratings and depressive symptoms, which may suggest a common network substrate.

In PWEs, self-esteem was considered moderate according to the SES scale. Self-esteem is a valuable aspect of the individual’s perception of his or her self-worth and whether he or she considers himself or herself capable, competent, and valuable, being related to mental health and psychological well-being, which can confirm our significant findings between lower self-esteem (feeling needy) and subjective memory impairment. Similar data have been described in healthy elderly people.^28^

There was no association between memory complaints and other clinical aspects of epilepsy, which suggests that multiple aspects, including psychological and neurobiological factors, may be related to the perception of impairment in cognition.

## LIMITATIONS OF THE STUDY

This study has some limitations. This was a cross-sectional study with a relatively small sample size from a single institution, and only cognitive screening tests were used. Our epilepsy clinic is in a university hospital, but it is not a tertiary epilepsy center. Studies with larger sample size are required to assess the impact of the findings of this study.

In PWEs, memory complaints were frequent, and there was a relationship with a deficit in cognitive assessment and educational level, the presence of depression, and low self-esteem.
